# Gut Microbiota Dysbiosis in Endometriosis: A Potential Link to Inflammation and Disease Progression

**DOI:** 10.3390/ijms26115144

**Published:** 2025-05-27

**Authors:** Alexandra Irma Gabriela Baușic, Francesca Scurtu, Andrei Manu, Daniela Roxana Matasariu, Elvira Brătilă

**Affiliations:** 1 Department of Obstetrics and Gynecology, “Carol Davila” University of Medicine and Pharmacy, 020021 Bucharest, Romania; alexandra.bausic@umfcd.ro (A.I.G.B.); francesca.scurtu@umfcd.ro (F.S.); andrei.manu@umfcd.ro (A.M.); elvira.bratila@umfcd.ro (E.B.); 2 Department of Obstetrics and Gynecology, “Prof. Dr. Panait Sîrbu” Obstetrics and Gynecology Hospital, 060251 Bucharest, Romania; 3 Department of Obstetrics and Gynecology, Obstetrics and Gynecology Filantropia Hospital, 011132 Bucharest, Romania; 4 Department of Obstetrics and Gynecology, Doctoral School, “Carol Davila” University of Medicine and Pharmacy, 020021 Bucharest, Romania; 5 Department of Obstetrics and Gynecology, University of Medicine and Pharmacy “Grigore T. Popa”, 700111 Iasi, Romania; 6 Department of Obstetrics and Gynecology, Cuza Vodă Hospital, 700038 Iasi, Romania

**Keywords:** endometriosis, intestinal microbiota, microbiome, endometriosis biomarkers

## Abstract

Endometriosis is a complex gynaecological disorder characterised by the presence of endometrial-like tissue outside the uterus, leading to chronic inflammation, pain, and infertility. Recent research suggests that gut microbiota may play a crucial role in the pathogenesis and progression of endometriosis by modulating immune responses and oestrogen metabolism. This study investigates the intestinal microbiota composition in women with endometriosis and its potential as a disease diagnosis and severity biomarker. Stool samples from nine patients diagnosed with endometriosis were analysed using the GI Effects^®^ Comprehensive Stool Profile test. The tests revealed significant dysbiosis, particularly an altered Firmicutes/Bacteroidetes ratio and increased levels of Bacteroidetes. Inflammatory markers, including β-glucuronidase and secretory IgA, were also elevated, suggesting a potential link between gut microbiota and systemic inflammation in endometriosis. While our findings align with previous studies, further research with larger cohorts is necessary to validate these observations. Understanding the role of the microbiome in endometriosis could open new avenues for noninvasive diagnostic tools in endometriosis and microbiota-targeted therapies.

## 1. Introduction

The widely disputed health condition known as endometriosis (EM) occurs when endometrial cells are present in ectopic locations: ovaries, adnexae, pelvic region, uterine ligaments, and rectovaginal septum [1].

Infertility, dysmenorrhea, dyspareunia, and gastrointestinal symptoms are possible side effects that might negatively impact an individual’s capacity to conceive and overall quality of life. EM affects as many as 10% to 15% of women of reproductive age, according to earlier research, and the incidence rate is rising annually [1,2]. The pathogenesis of EM remains difficult to reliably explain even after three centuries of research, despite the publication of several theories on its development [2,3].

Numerous studies have found a range of potential endotoxins in the peritoneal cavity of EM patients, which may control the pro-inflammatory response and encourage the development of endometriosis [3,4,5,6]. As a result, some researchers have suggested the “bacterial contamination hypothesis” as the primary cause of EM [7]. Accordingly, limited information is known about intrauterine microbial colonisation.

The 16S rRNA gene amplicon new generation sequencing technology was used to explain that the female reproductive system, which includes the cervical canal, uterus, fallopian tubes, and peritoneal fluid, continually harboured a unique microbial population [8].

The microbiota genome has been sequenced over time, with a focus on populations that colonise the vagina, digestive system, nasal passages, oral cavity, and skin. The genetic diversity of the microbiota within a particular ecosystem or environment is represented by the microbiome [9]. A microbiota imbalance caused by a rise in pathogenic species or a fall in protective species is referred to as dysbiosis [10].

The microbiota influences immune modulation and the development of several inflammatory disorders. It is widely known that the gut microbiota inhibits bacterial translocation, which can impair immunological homeostasis, cause low-grade systemic inflammation, and damage the gastrointestinal epithelial barrier. The immune system’s ability to tolerate commensals and self-antigens while remaining susceptible to infection is guaranteed by immunological homeostasis [10,11,12].

Significant differences in composition between the human microbiota and autonomous microbes have been found in a variety of research [13]. Consequently, commensal expansion of the microbiota has led to the selection of organ-specific bacterial communities. Each of the communities that comprise the microbiome has distinct characteristics. For instance, patients with bacterial vaginosis frequently have an increase in the number of bacteria at both the intestinal and vaginal levels, whereas healthy women have diversified intestinal microbiota free of high levels of pathogenic species [13,14].

In endometriosis patients, intestinal, vaginal, and uterine dysbiosis contributes to immune response modulation, changes in oestrogen metabolism, and disease aggravation. Dysbiosis weakens the intestinal epithelial barrier, increasing cell permeability and bacterial efflux, and influencing the pathways by which bacteria enter the peritoneal fluid by reducing epithelial junctions [15]. Bacteria enter the lymphatic circulation through the activation of follicular dendritic cells in the intestinal wall’s lymph nodes [16]. Bacteria also enter the bloodstream directly.

The microbiota and endometriosis have a reciprocal interaction. Immunological function is suppressed, intestinal barrier permeability rises, and pathogen defences are diminished when intestinal dysbiosis is present. The intestinal microbiota’s regular processes of nutritional absorption and synthesis, intestinal mucosal integrity maintenance, pathogen defence, immune system development, and control under inflammatory circumstances are all disrupted by an imbalance in the microbiota [15].

A persistent inflammatory state is maintained and the growth of endometriotic implants is accelerated by uncontrolled immune cell activation and pro-inflammatory cytokine release. The capacity of macrophages to phagocytise ectopic endometrial tissue is diminished, giving them a unique character. Inhibiting stomach acid production and intestinal motility, intraperitoneally released cytokines reduce lactobacilli species and promote the growth of gram-negative bacterial species [12].

When examining the relationship between endometriosis and the gut microbiome, multiple microbial ecosystems must be considered. Prior studies have reported associations between endometriosis and elevated levels of various microorganisms, including *Bacteroides*, *Clostridia*, *Prevotella*, *Proteobacteria*, *Firmicutes*, *Bacteroidetes*, *Enterobacteriaceae*, *Brucellaceae*, *Klebsiella*, *Gardnerella*, *Shigella*, *Streptococcus*, *Actinobacteria*, *Cyanobacteria*, *Fusobacteria*, *Paraprevotella*, *Escherichia coli*, *Odoribacter*, *Veillonella*, and *Ruminococcus* [17,18,19,20,21,22,23].

The aim of this study was to investigate whether the gut microbiota and enzymatic activity profiles in individuals with endometriosis show altered patterns when compared to established reference ranges. This exploratory analysis was conducted using stool samples from a cohort of nine patients diagnosed with endometriosis, and microbial and enzymatic data were compared to normative values provided by clinically validated laboratory testing standards.

## 2. Results

The intestinal microbiota test confirms the existence of commensal gut microbiota, which is prevalent in species reported in the literature. The following pattern of inflammation-related dysbiosis was discovered in the results presented. The GI Effects^®^ Comprehensive Stool Profile test analyses the abundance and diversity of the seven major bacterial phyla in commensal species with the levels considered typical in the standard population, thus we did not create a control group to compare the results [24]. This would suggest more substantial variations in the gut microbiota structure in individuals with endometriosis. The presence of seven commensal microbial taxa (*Bacteroidetes*, *Firmicutes*, *Actinobacteria*, *Proteobacteria*, *Euryarcaeota*, *Fusobacteria*, and *Verrucomicrobia*), *Clostridium* spp., the Firmicutes/Bacteroidetes ratio, and inflammatory markers (secretory IgA in faeces, calprotectin, eosinophil cationic protein A(EPX), and β-glucuronidase) were all investigated.

In contrast to the standard population, overall commensal abundance refers to the total number of commensal microorganisms observed. It is common to observe reduced levels of commensal bacteria after antimicrobial therapy or in diets lacking in fibre and/or foods high in prebiotics. This might indicate the need to nourish the microbiota. Conversely, higher commensal abundance overall may indicate the use of probiotics or even bacterial overgrowth.

Anything below 10% is considered a possible gut microbiome shortage, and anything beyond 10% is considered a potential overgrowth. The usual range is between −10% and 10%. Five of the nine patients had an overabundance of gut microbiome species, one patient had a lack of typical gut commensal species, and three patients had normal gut microbiota. The quantity of each of the seven major bacterial phyla was compared to a healthy group using relative abundance. This might point to more significant differences in the patients’ gut microbiota profiles. Between −25% and 25% is thought to be the typical range for the number of bacterial phyla in the gut microbiota.

**Figure 1:**
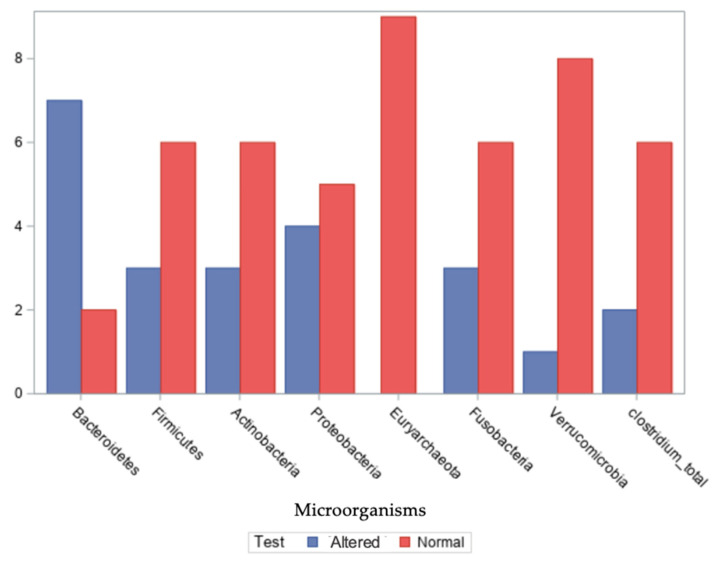
Frequency of test results for microorganisms in the intestinal microbiota of patients.

Seven commensal microbial species—*Bacteroidetes*, *Firmicutes*, *Actinobacteria*, *Proteobacteria*, *Euryarcaeota*, *Fusobacteria*, and *Verrucomicrobia*—were investigated in the stool samples of the nine patients, along with the detection of *Clostridium* spp. (Figure 1). Values for the number of bacterial phyla in the gut microbiota are presented in Supplementary Table S2.

Regarding the *Bacteroidetes phylum*, seven out of nine patients exhibited altered relative abundances, with values ranging from −15% to 56%. For the *Firmicutes phylum*, altered levels were observed in three out of nine patients, with values ranging from −55% to 39%, −25% to 25% being the normal range for the number of bacterial phyla in the gut microbiota. In the case of the *Actinobacteria phylum*, three patients showed deviations from typical levels, with a range of −55% to 10%. For the *Proteobacteria phylum*, four out of nine patients exhibited distinct compositional profiles, with values ranging from −50% to 30%.

Regarding the *Euryarchaeota phylum*, all nine patients had values within the expected reference range, spanning 0% to 23%. For the *Fusobacteria phylum*, three out of nine patients showed altered relative abundances, with values between 0% and 30%.

In the case of the *Verrucomicrobia phylum*, one patient exhibited a notable deviation, with values across the group ranging from −30% to 22%. Finally, regarding *Clostridium* spp. presence in stool samples, distinct patterns were identified in two out of nine patients.

**Figure 2:**
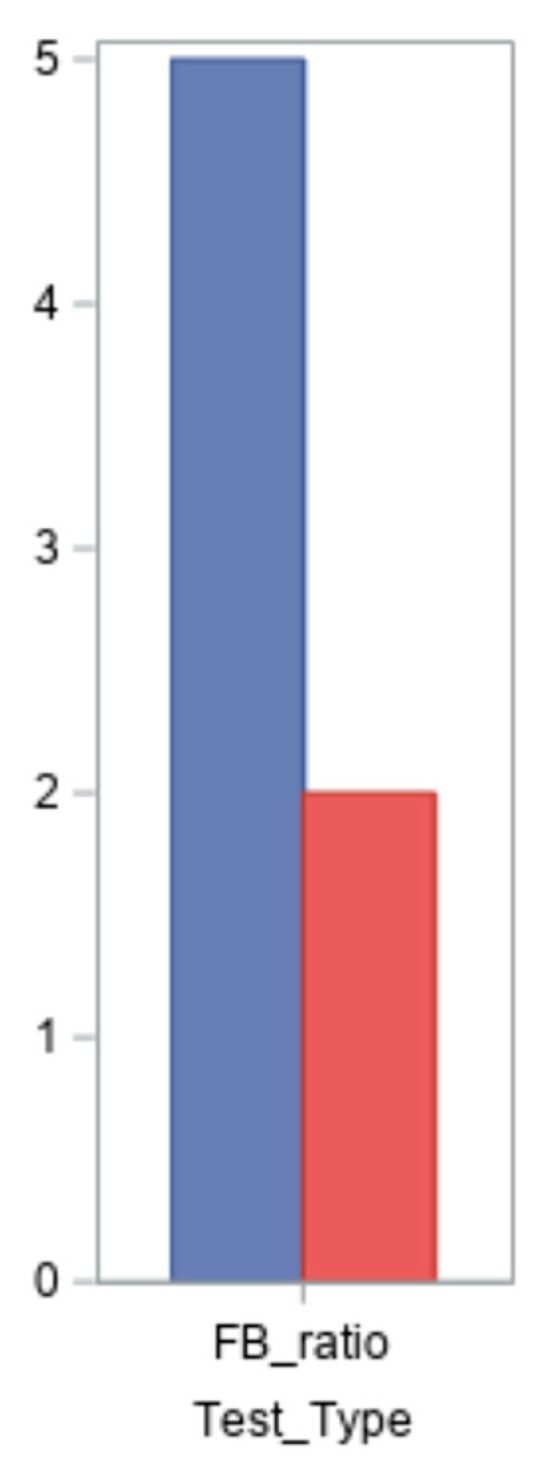
Results of the Firmicutes/Bacteroidetes clinical test in the patient group.

An imbalance in the intestinal microbiota is indicated by an increase or reduction in the Firmicutes/Bacteroidetes ratio (FB ratio), which is cited in the literature as a key indicator of intestinal dysbiosis [21]. This ratio’s value is less than 1.5 in the normal population. Seven of the nine patients had their ratios evaluated; the findings varied from 0.5 to 35, with two normal values and five altered values (Figure 2).

In this study, the Firmicutes/Bacteroidetes (F/B) ratio was reported for seven out of nine participants. For two participants, the ratio could not be calculated due to incomplete data provided by the external laboratory, specifically the absence of quantifiable values for one or both phyla. While these missing values did not significantly impact the overall interpretation of findings, we acknowledge this as a limitation of the dataset. Future studies will prioritise more standardised and comprehensive data collection to ensure consistency across all samples.

**Figure 3:**
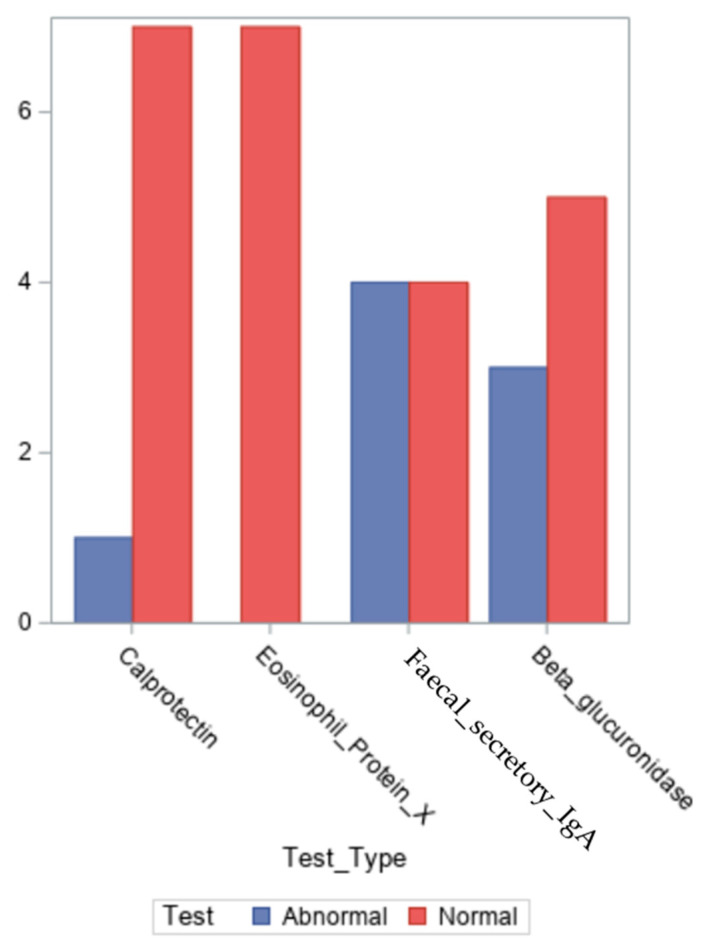
Results of the clinical tests for enzymes detected in the stool of patients in the group.

As illustrated in Figure 3, we measured the levels of the inflammatory indicators calprotectin, eosinophil cationic protein X, secretory IgA in faeces and β-glucuronidase. The values for all the inflammatory markers are presented in Supplementary Table S1. 

The intestinal epithelium and some bacterial species secrete the enzyme β-glucuronidase, which is vital for digestion, particularly for the breakdown of complex carbohydrates and the detoxification of oestrogen and other environmental pollutants [25]. In premenopausal women, higher β-glucuronidase levels have been linked to decreased faecal elimination of oestrogens and higher levels of circulating oestrogen [25]. Reduced bacterial diversity, extreme diets, or probiotic and/or prebiotic treatment are all associated with low β-glucuronidase activity, which is also a sign of aberrant metabolic activity within the intestinal microbiota [21,25].

Nine bacterial species that produce β-glucuronidase were found by Dabek et al. [20] in the gut microbiota: *Bacteroides ovatus*, and members of the *Firmicutes* phylum’s Lachnospiraceae family: *Faecalibacterium prausnitzii* (family Oscillospiraceae of the Phylum *Bacillota*) and *Roseburia intestinalis* and *hominis*. High levels of β-glucuronidase appear to be most commonly linked to the *Firmicutes* phylum [26].

The β-glucuronidase level in the test is 368–6266 U/g, which is regarded as normal. The median value for our patients was 2302.5 U/g, and the average value was 2564.1 U/l (numbers ranging from 0 to 7206 U/g).

Faecal secretory IgA, calprotectin, and cationic protein of eosinophils X are examples of inflammatory indicators that indicate the activation of different immune cells in the immune response pathways.

Faecal secretory IgA serves as an immunological barrier and is the first line of defence protecting the mucosa of the gastrointestinal tract. Its presence is necessary for the digestive system to function normally [25]. It is crucial in regulating the effects of bacteria, parasites, and viruses on the gut environment. Amounts in the range 0–680 mcg/g are regarded as normal, while 608–2040 mcg/g are regarded as borderline [25]. Intestinal permeability, autoimmune disorders, coeliac disease, gastrointestinal infections, and inflammatory bowel disease are all associated with low levels of secretory IgA. In the setting of intestinal microbiota dysbiosis, elevated secretory IgA levels are linked to enhanced intestinal mucosal immune defence [25].

According to the test, secretory IgA levels between 0 and 2040 mcg/g are considered normal. Four of the nine patients had values that were greater than the upper limit of normal, indicating the existence of intestinal dysbiosis. Four of the patients had normal measurements and for one patient the result has not been reported. With values ranging from 545 to 7500 mcg/g, their median was 1991.5 mcg/g, while their average was 2492.7 mcg/g, for all the eight patients.

Another inflammatory marker is faecal calprotectin, a protein belonging to the S-100 family that mostly localises in neutrophils and has the function of binding calcium and zinc. As a result of neutrophil migration into the gastrointestinal system during an inflammatory condition, calprotectin is found in faeces [27]. According to the test, a calprotectin level of less than 50 mcg/g is considered normal. Only one patient had an altered calprotectin value, while the other seven out of nine patients had normal readings and for one patient the result has not been reported. They ranged from 16 to 59 mcg/g, with a median of 17 mcg/g and a mean of 22 mcg/g.

Stool containing the EPX indicates eosinophilic activity in the gastrointestinal system. An inflammatory disorder in the gastrointestinal tract, such as Crohn’s disease, ulcerative colitis, chronic diarrhoea, chronic gastro-oesophageal reflux, or intestinal infection, is indicated by abnormally high levels of the eosinophil X protein [28]. According to the test, a level of less than 2.7 mcg/g is regarded as normal for EPX [28]. Seven out of nine patients had readings that were within normal limits. They had a median of 0.1 mcg/g (numbers between 0 and 0.8 mcg/g) and a mean of 0.25 mcg/g.

We examined each of the nine patients’ results on the Genova Diagnostics proprietary inflammation-associated dysbiosis (IAD) score [24,28]. The average IAD score had a positive correlation with secretory IgA, faecal calprotectin, and EPX and a negative correlation with commensal abundance [28]. Clinical research using the Genova database of inflammatory bowel disease patients has validated the score [24]. The score looks into whether inflammation-associated dysbiosis results from inflammation or a cause of it [28].

In the test performed, the normal level of the IAD score is 60 or below. Values below 60 are seen to have a minimal probability of having an inflammatory status, while all values over 60 are regarded as high and linked to inflammation. Three of the nine individuals had altered readings that exceeded the upper limit of normal, whereas the other six had normal outcomes. Their median (values between 0 and 74) was 10, and their mean was 28.44. Three patients presented values considered to be high, suggesting the existence of intestinal dysbiosis.

We sought to determine whether the likelihood of observing altered enzyme levels or distinct microbial profiles in the endometriosis patient population is greater than the likelihood of observing values within reference ranges, based on findings reported in the specialised literature.

Using findings from the specialised literature, we looked into whether the proportion of an altered test (*p* abnormal) for the eight commensal microbial species (*Bacteroidetes*, *Firmicutes*, *Actinobacteria*, *Proteobacteria*, *Euryarcaeota*, *Fusobacteria*, *Verrucomicrobia*, and *Clostridium* spp.), the Firmicutes/Bacteroidetes ratio, inflammatory markers (calprotectin, β-glucuronidase, eosinophil cationic protein A, secretory IgA in faeces), and the inflammation-associated dysbiosis score was greater than 1/2 (pure chance) in patients with endometriosis.

We wanted to investigate whether women with endometriosis are more likely to have altered results on a test that identifies the presence of *Bacteroidetes*, compared to normal results. That is, the probability *π* that the test for the abundance of the phylum *Bacteroidetes* is altered in the population of women with endometriosis is greater than chance (*π*_anormal_ > 0.5).

We had access to a group of nine patients with endometriosis in whom we tested the relative abundance of the phylum *Bacteroidetes*. The number of times an altered test for *Bacteroidetes* occurs in our sample of nine patients with endometriosis is modelled as having a binomial distribution Binomial (9, *π*).

We will statistically test the following hypotheses—null versus alternative.

H_0_: *π*_anormal_ ≤ 0.5 (a “clinical test” is not more likely to be altered than normal).

H_a_: *π*_anormal_ > 0.5.

Because the sample is very small (*n* = 9, 8, or 7), we set the significance level at *α* = 0.10 (90% confidence). For *Bacteroidetes*, the one-sided right exact binomial test with *p*-value = Prob (number of altered *Bacteroidetes* tests ≥ 7) = 0.090, which is less than 0.10 = *α*, rejects the null hypothesis. There is enough evidence in the data to reject the statement that the *Bacteroidetes* test has a probability less than or at least equal to 0.5 of being normal. Seven of the nine patients had an altered test for *Bacteroidetes*.

The one-sided Clopper–Pearson 90% confidence intervals are (0.51, 1.00) [29].

They lie completely to the right of the value 0.5 (chance), which also indicates that the *Bacteroidetes* test is more likely to be altered in the population of women with endometriosis. This suggests that there is sufficient evidence in our sample to show that the proportion of altered clinical tests is greater than pure chance (1/2).

For the other seven commensal microbial species (*Firmicutes*, *Actinobacteria*, *Proteobacteria*, *Euryarcaeota*, *Fusobacteria*, *Verrucomicrobia*, and *Clostridium* spp.), for the Firmicutes/Bacteroidetes ratio and for the inflammatory markers (calprotectin, β-glucuronidase, eosinophil cationic protein X, faecal secretory IgA), the one-sided right exact binomial test yields a *p*-value ≥ 0.10, thus we fail to reject the null hypothesis that a “clinical test” has a probability less than or at least equal to 0.5 of being altered in the population of women with endometriosis. We failed to reject the null hypothesis, suggesting that there is insufficient evidence in our cohort to reject the statement that the proportion of altered tests is equal to 1/2 (pure chance).

The full results of the statistical test are presented in Table 1. With only nine patients enrolled in the study, the results cannot be transferred to the population level, which is a limitation of our analysis. Continuing the study by enrolling more patients with endometriosis may lead to more precise results, comparable to those studied in the literature.

## 3. Discussion

Invasive procedures are necessary for a conclusive diagnosis of endometriosis because its onset can be difficult to notice (laparoscopic excision of endometriosis lesions and confirmation of the histological diagnosis). No biological markers are both sensitive and specific in the present diagnostic method used in clinical practice. This deficiency causes a delay in the diagnosis and treatment of the illness, which has a major effect on women’s quality of life [30].

Therefore, the clinical diagnosis and therapy of endometriosis greatly depend on the quest for non-invasive biomarkers.

Women with endometriosis have been found to have specific diagnostic indicators in their menstrual blood, urine, or serum. With the benefits of easy, quick, and non-invasive detection, the use of these markers is becoming a crucial avenue for illness diagnosis. There are known links between endometriosis and the microbial health of the vaginal and gastrointestinal tracts [31].

Endometriotic implants promote bacterial pathogens’ persistence through oestrogen metabolism changes, demonstrating the reciprocal link between endometriosis and the gut microbiota. By favouring a chronic inflammatory and hyperoestrogenic environment, the microbiota plays a role in the development of endometriosis [32].

Investigating the connection between endometriosis and the gut microbiota requires taking into account a variety of microbial habitats. The elevated levels of several microorganisms in the gut microbiome, including *Bacteroides*, *Clostridia*, *Prevotella*, *Proteobacteria*, *Firmicutes*, *Bacteroidetes*, *Enterobacteriaceae*, *Brucellacea*, *Klebsiella*, *Gardnerella*, *Shigella*, *Streptococcus*, *Cyanobacteria*, *Facteria*, *Sacchainobacteria*, *Paraprevotella*, *Escherichia coli*, *Odoribacter, Veillonella,* and *Ruminococcus*, appear to be linked to endometriosis [17,19,33,34,35,36,37]. Our analysis confirmed the findings reported in the specialised studies by examining the faecal microbiota of endometriosis patients.

Patients who participated in the study had high levels of bacteria from the phylum *Bacteroidetes* in their intestinal microbiota; seven of them had altered values above the threshold value deemed normal, which is consistent with the findings of studies by Svensson, Chadchan, and Shan [33,36,37]. The results of Shan [36] indicate the existence of the Fusobacteria Phylum. The results of Yuan and Shan [17,36] suggest that the stool samples of the patients contained *Proteobacteria, Actinobacteria*, and *Firmicutes Phylum*.

Within the normal values range, the patients’ intestinal microbiome exhibited the lowest levels from the phyla *Euryachaeota* and *Verrucomicrobia*. Although there is no proof that *Euryachaeota* is present or associated with endometriosis, Shan found the *Verrucomicrobia* Phylum in stool samples from endometriosis patients [36].

There is no evidence in the literature linking the onset of endometriosis to the absence of any bacterial species. According to Shan’s (2020) research, patients with endometriosis had a higher Firmicutes/Bacteroidetes ratio. Numerous studies have used this ratio as a dysbiosis biomarker [36].

*Actinobacteria*, *Cyanobacteria*, *Saccharibacterium*, *Fusobacterium*, *Bifidobacterium*, *Blautia*, *Dorea*, *Streptococcus*, and *Acidobacteria* were found to be abundant in the group of endometriosis patients in Shan’s study, while *Tenericutes*, *Lachnospira*, and *Eubacterium* were found to be decreased [36]. The investigation suggested a link between elevated levels of oestrogen and inflammatory markers (serum IL-8) and intestinal dysbiosis in endometriosis patients [36].

Yuan (2018) found that intestinal dysbiosis occurred 42 days after the persistence of endometriotic lesions in a group of laboratory mice whose endometrial tissue was implanted in the peritoneal cavity [17]. *Firmicutes* and *Actinobacteria Phylum* were the species found in the intestinal microbiota, whereas *Bacteroidetes* was found in the control group [17]. The endometriosis group had a greater Firmicutes/Bacteroidetes ratio, which is a significant indicator of dysbiosis. According to this study, endometriosis and the microbiota have a mutually beneficial connection.

Five of the patients in our cohort had Firmicutes/Bacteroidetes ratios beyond the usual range of less than 1.5, with values ranging from 0.5 to 35. Intestinal dysbiosis is indicated by an increase or reduction in the ratio, which is impacted by a number of variables, including age, weight, diet, antibiotics, supplements, and physical activity. The Firmicutes/Bacteroidetes ratio in endometriosis is not well documented in the literature; the most pertinent research is being carried out on mice [17]. While it is possible that a drop in the ratio might be interpreted as a sign of dysbiosis, pertinent research has demonstrated an increase in the Firmicutes/Bacteroidetes ratio in endometriosis patients [17,19].

Regarding beta-glucuronidase activity, Dabek identified nine bacterial strains with beta-glucuronidase activity after studying 40 distinct strains that belong to the prominent bacterial groups in the gut microbiota: *Roseburia intestinalis*, *Roseburia hominis*, and *Faecalibacterium prausnitzii* (family *Oscillospiraceae* of the *Phylum Bacillota*) are all members of the *Lachnospiraceae* family, which includes *Bacteroides ovatus* [20].

*Roseburia hominis*’s notable effect on beta-glucuronidase activity raises the possibility that certain intestinal microbiota components may exhibit varying degrees of enzymatic activity based on the colon’s exposure to glycosides, which is impacted by dietary patterns. The phylum Firmicutes appears to be most closely linked to elevated beta-glucuronidase levels. Dysbiosis also impacts oestrogen metabolism by releasing beta-glucuronidase, speeding up oestrogen deconjugation, and raising the amount of free circulating oestrogen [25,36].

The three inflammatory markers under investigation—faecal calprotectin, EPX, and secretory IgA in faeces—show that different immune cells are activated in immune response pathways.

Because of its strong correlation with endoscopic activity scores, elevated calprotectin levels have been linked to inflammation in inflammatory bowel diseases, such as Crohn’s disease and ulcerative colitis. It is also a sensitive marker for tracking intestinal disease activity and can be used as an alternative to colonoscopy [27]. The IAD score looks at whether inflammation-related dysbiosis is a cause or an effect of inflammation. In patients with coeliac disease or inflammatory bowel disease, the IAD score has been effectively applied and shown to have a good correlation with intestinal inflammation biomarkers [28].

Given the possibility that an inflammatory intestinal microbiome pattern precedes the rise in inflammatory markers and promotes the growth of endometriotic implants, this score may be used to diagnose intestinal inflammation linked to endometriosis, particularly intestinal endometriosis.

Other causes should be looked into if there is a low IAD score accompanied by high inflammatory markers, which suggests that the gut microbiota may not be involved in the inflammatory state. The significance of a high IAD score with normal inflammatory markers requires longitudinal research [28].

In Chen’s work, the IAD score—which excludes EPX and secretory IgA levels—is derived from an algorithm that uses information about the gut microbiota, faecal beta-glucuronidase, and faecal calprotectin levels. Faecal calprotectin, EPX, and IgA levels were directly correlated with the mean IAD score, while commensal abundance was inversely correlated with it [28].

In the group of patients with inflammatory bowel illness, Chen’s study found positive correlations with high levels of EPX, secretory IgA, and calprotectin [28]. Compared to the healthy control group, the patients with Crohn’s disease and ulcerative colitis had considerably greater levels of calprotectin, EPX, and an IAD score.

However, there was no discernible statistical difference in commensal abundance across all groups. In terms of inflammatory biomarkers and IAD score, the group of patients with coeliac disease and irritable bowel syndrome was identical to the control group [28].

In order to forecast a condition of dysbiosis linked to intestinal inflammation, the study makes the assumption that the IAD score is far more useful than determining the intestinal microbiota [28].

Due to an increasing awareness of the various roles the gut microbiota plays in various diseases, research on the gut microbiome has advanced to the point where it can be selectively managed to cure endometriosis or, in part, its symptoms [21]. The general objective is to replenish the gut with commensal microorganisms to restore equilibrium, while the specific commensal species targeted may differ based on the condition [38]. The idea behind microbiome-based therapy is to restore the positive activities of the microbiome, such as bolstering the intestinal mucosal barrier, boosting resistance to intestinal colonisation, and re-establishing normal interactions between the immune system and the microbiota [38].

Dysbiosis is linked to an imbalance in patients’ microbiota when compared to a healthy population, which can cause various diseases to develop or worsen [38].

Since there are not many studies on the topic, the primary strength of the current study is the data gathered from the literature on the intestinal microbiota composition in both humans and mice, in both the endometriosis and control groups. Additionally, we were able to contrast these results with information gathered from study participants.

The intestinal microbiota is highly significant and offer several opportunities for endometriosis therapy, diagnosis, and prevention [39].

Our investigation was limited by the short number of data standardisation works accessible in the literature, the small number of patients who were enrolled, and the inability to extrapolate the results to the population level. The lack of a healthy control group is a second drawback. More endometriosis patients could be added to the study to generate more accurate findings that are comparable to those found in previous research.

One key limitation of this study is the absence of a control group. Due to logistical and resource constraints, we were unable to recruit a cohort of healthy individuals or disease controls without endometriosis for direct comparison. Instead, we relied on established reference ranges from the literature and clinical laboratory standards to contextualise our findings. While this approach offers preliminary insights into potential microbial and enzymatic alterations in individuals with endometriosis, it limits the strength of inferences that can be drawn about disease-specific patterns. The inclusion of a matched control group would have provided a more robust comparative framework, helping to distinguish findings unique to endometriosis from those that may occur more broadly in the population. We recognise this as a limitation of the current study and plan to address it in future research through larger sample sizes and the incorporation of appropriate control groups.

Investigating relationships between the gut microbiome profile, stool biomarkers, and the existence of endometriosis lesions utilising biomarkers and the IAD score—which is now used specifically for inflammatory bowel diseases—is one of the study’s strong points.

## 4. Materials and Methods

This study was approved by the Medical Ethics Committee of “Prof. Dr. Panait Sîrbu” Obstetrics and Gynecology Hospital in Bucharest, Romania. Informed consent was completed for all subjects enrolled in the study. The nine patients involved in this study were aged 27–38 years (33.55 years old on average), expressed chronic pelvic pain symptomatology (100% dysmenorrhea, 55.55% dyspareunia, and 77.77% gastrointestinal symptoms) and were diagnosed by transvaginal ultrasound (TVS) or magnetic resonance imaging (MRI) with endometriosis.

Regarding the existence of gastrointestinal symptoms (transit disorders—constipation/diarrhoea, dyschezia, abdominal bloating) and the imaging identification of endometriotic lesions at the recto-sigmoid level, 6 out of 9 patients presented images suggestive of recto-sigmoid endometriotic nodules on pelvic MRI images.

All research participants had surgical treatment for endometriosis after being evaluated using TVS and a 3.0 Tesla nuclear magnetic resonance system. Histopathological analysis was used to confirm the final diagnosis of endometriosis. Genova Diagnostics’ particular GI Effects^®^ Comprehensive Stool Profile test was used to analyse stool samples and evaluate the microbiome’s condition [24]. This test panel allowed us to gather data on a number of inflammatory indicators, including calprotectin, β-glucuronidase, eosinophil cationic protein A, faecal secretory IgA, and seven commensal bacteria species identified by PCR and microscopy.

Descriptive statistics for categorical variables were expressed as frequencies and percentages of altered tests.

## 5. Conclusions

A topic of scientific interest, a unique bilateral connection between microbiota and endometriosis has started to be reported in specialised studies. According to laboratory testing, the microbiome profiles of individuals with and without endometriosis can differ significantly. The diagnosis of deep infiltrative endometriosis, particularly endometriosis with rectosigmoid infiltration, may also be made using these three inflammatory biomarkers and the IAD score.

If the composition of the gut microbiota and endometriosis are related, how would one identify which is the cause—different microbiota causing endometriosis and changed immune function, or an altered immune response causing endometriosis and different microbiota?

Since the studies listed in the bibliography do not employ the same microbial tests or identify the same bacterial species, we can draw the conclusion that there is a lack of consistency about the specific microbiota that we should look at in patients with endometriosis.

The study confirms the findings in the previously mentioned literature, but more investigation and data analysis on a larger patient population, utilising a more exacting methodology and standardising clinical tests, are required to elucidate the role of the microbiome in the pathophysiology of endometriosis.

When paired with imaging and clinical assessment, the composition of the gut microbiota can be utilised as a screening test for endometriosis. Determining the gut microbiota may be a great screening tool to direct a patient to a gynaecologist who specialises in endometriosis if the patient has chronic pain and gastrointestinal symptoms that do not improve with anti-inflammatory treatment but has not yet received an endometriosis diagnosis. Examining the potential of the microbiome as a noninvasive endometriosis diagnosis technique will be beneficial.

## Figures and Tables

**Table 1:** Statistical results.

Clinical Test	N	Number of Clinical Test		Proportion of Altered Tests Prop = #Altered/#Total	90% One-Sided Confidence Interval Clopper–Pearson (Exact)	Exact Binomial Test (Distribution Binomial (N,*π*)) Ho: *π* ≤ 0.5 vs. Ha: *π* > 0.5 *p*-Value
		Altered	Normal			
Phylum						
Bacteroidetes	9	7	2	0.78	0.51–1.00	0.090
Firmicutes	9	3	6	0.33	0.13–1.00	0.910
Actinobacteria	9	3	6	0.33	0.13–1.00	0.910
Proteobacteria	9	4	5	0.44	0.21–1.00	0.746
Euryarcaeota	9	0	9	0.00	0.00–1.00	1.000
Fusobacteria	9	3	6	0.33	0.13–1.00	0.910
Verrucomicrobia	9	1	8	0.11	0.01–1.00	0.998
Clostridium spp	8	2	6	0.25	0.07–1.00	0.965
Firmicutes/Bacteroidetes ratio	7	5	2	0.71	0.40–1.00	0.227
Enzymes						
Calprotectin	8	1	7	0.13	0.01–1.00	0.996
Eosinophil Protein X	7	0	7	0.00	0.00–1.00	1.000
Faecal Secretory IgA	8	4	4	0.50	0.24–1.00	0.637
Betaglucuronidase	8	3	5	0.38	0.15–1.00	0.856
IAD score	9	3	6	0.33	0.13–1.00	0.910

N—total number of patients, *π* = prob (altered test) in population.

## Data Availability

The original contributions presented in this study are included in the article/Supplementary Materials. Further inquiries can be directed to the corresponding author(s).

## Supplementary Materials

The following supporting information can be downloaded at: https://www.mdpi.com/article/10.3390/ijms26115144/s1.
