# Supplementary material for: Gut Microbiota Dysbiosis in Endometriosis: A Potential Link to Inflammation and Disease Progression
Source: Int J Mol Sci. 2025 May 27;26(11):5144. doi: 10.3390/ijms26115144 (PMC12153989; doi:10.3390/ijms26115144)
Supplement: Supplementary file 1 [file ijms-26-05144-s001.zip › ijms-3631367-supplementary.pdf]

International Journal of
Molecular Sciences

MDPI

Supplementary tables

Table S1. Values for the inflammatory markers for all the patients

PATIENTS	CALPROTECTIN	EPX	FAECAL SECRETORY IGA	B-GLUCU-RONIDASE
NORMAL	16 to 59 mcg/g	<2.7 mcg/g	0 - 2040 mcg/g	368–6266 U/g
**1**	59	0,8	7500	518
**2**	17	0	1049	6440
**3**	16	0	545	786
**4**	17	0,7	866	1556
**5**	16	0	643	0
**6**	16	0,2	2951	3049
**7**	18	-	-	-
**8**	17	0,1	3614,3	7206
**9**	-	-	2774	5607

Table S2. Values for the number of bacterial phyla in the gut microbiota

**Phylum** / **Patients**	**Bacteroidetes (%)**	**Firmicutes (%)**	**Actinobacteria (%)**	**Proteobacteria (%)**	**Euryarchaeota (%)**	**Fusobacteria (%)**	**Verrucomicrobia (%)**	**Clostridium spp** (CFU/g faeces0
1	30%	-5%	-50%	5%	0%	30%	0%	2.8 x 10^9
2	30%	5%	-45%	30%	20%	20%	-5%	5.6 x 10^9
3	30%	5%	5%	30%	0%	28%	2%	4.9 x 10^9
4	30%	5%	2%	30%	15%	0%	-15%	1.6 x 10^9
5	30%	-55%	-55%	-50%	23%	0%	0%	8.7 x 10^8
6	-3%	3%	10%	15%	0%	30%	-30%	3.8 x 10^5
7	56%	30%	1%	5%	0%	0%	5%	0

8	43,80%	38,90%	0,20%	16,90%	20%	0%	0%	4,3 x 10^8
9	-15%	-5%	-7%	2%	0%	0%	22%	0
